# Interleukin-2 improves tumour response to DNP-modified autologous vaccine for the treatment of metastatic malignant melanoma

**DOI:** 10.1038/sj.bjc.6601563

**Published:** 2004-02-17

**Authors:** M Lotem, E Shiloni, I Pappo, O Drize, T Hamburger, R Weitzen, R Isacson, L Kaduri, S Merims, S Frankenburg, T Peretz

**Affiliations:** 1 Sharett Institute of Oncology, Hadassah University Hospital, Jerusalem 91120, Israel; 2 Department of Surgery B, Carmel Medical Center, Haifa 34362, Israel; 3 Department of Surgery A, Assaf Harofe Medical Center, Zeriffin 70300, Israel; 4 Department of Oncology, Sheba Medical Center, Ramat Gan 52662, Israel; 5 Department of Oncology, Shaarei Zedek Medical Center, Jerusalem 91031, Israel; 6 Department of Dermatology, Hadassah University Hospital, Jerusalem 91120, Israel

**Keywords:** autologous melanoma vaccine, interleukin-2, metastatic melanoma

## Abstract

This paper is a report of response rate (RR) and survival of 34 metastatic melanoma patients who received a dinitrophenyl (DNP)-modified autologous melanoma cell vaccine. In all, 27 patients started the vaccine as a primary treatment for metastatic melanoma and seven started it as an adjuvant, with no evidence of disease at the time, but had developed new metastases. Interleukin-2 (IL-2) was administered in 24 out of the 34 patients: 19 who progressed on vaccine alone and five who had the combination from start. Interleukin-2 was administered in the intravenous, bolus high-dose regimen (seven patients) or as subcutaneous (s.c.) low-dose treatment (17). Overall response for the entire group was 35% (12 patients out of 34), 12% having a complete response (CR) and 23% a partial response (PR). However, only two patients had tumour responses while on the vaccine alone, whereas the other 10 demonstrated objective tumour regression following the combination with IL-2 (two CR, eight PR), lasting for a median duration of 6 months (range 3–50 months). Of the 12 responding patients, 11 attained strong skin reactivity to the s.c. injection of irradiated, unmodified autologous melanoma cells. None of the patients with a negative reactivity experienced any tumour response. Patients with positive skin reactions survived longer (median survival – 54 months). The results suggest enhanced RRs to the combination of IL-2 and autologous melanoma vaccine. Skin reactivity to unmodified autologous melanoma cells may be a predictor of response and improved survival, and therefore a criterion for further pursuing of immunotherapeutic strategies.

Metastatic malignant melanoma is associated with grave prognosis, not affected significantly by the varying treatment schedules. Chemotherapy is usually the standard therapy, but it is associated with considerable adverse reactions, and with the exception of a minority of complete responders, most patients succumb to their disease without any increase of survival. The combination of chemotherapy with the biologic response modifiers interferon-*α* (INF*α*) and interleukin-2 (IL-2) is associated with slightly higher response rates (RRs), but this is achieved at the cost of more severe toxicity (Atzpodien *et al*, 2002). Interleukin-2 can also be administered alone. There is no agreed upon standard regimen for IL-2, but the generally accepted protocols include intravenous (i.v.), high-dose pulse therapy with 600–750 000 IU kg^−1^ dose^−1^ (Atkins *et al*, 2000), continuous i.v. administration of 18 × 10^6^ IU m^−2^ day^−1^ or less (Dillman *et al*, 1993), and the decrescendo protocols, starting at 18 × 10^6^ IU m^−2^ dose^−1^, and decreasing. In the large series of Atkins *et al* (2000), the overall response rate to high-dose IL-2 is 16%, with 6% achieving complete tumour regressions. Lower doses of IL-2 alone, either i.v. or subcutaneously (s.c.), are considered to yield responses only rarely (Vlasveld *et al*, 1994).

Melanoma vaccines could have been an attractive alternative approach, but that their effectiveness in metastatic disease is limited: the RRs range from 5 to 15%, and the positive responses are mainly limited to s.c. metastases (Mitchell *et al*, 1990; Morton *et al*, 1992; Eton *et al*, 1998). Yet, a much higher proportion of patients treated with melanoma vaccines develop definite immune reactions. Although in most cases these reactions fall short of being translated into clinical tumour regressions, improved survival has been repeatedly reported for those patients who develop antimelanoma immune responses (Bystryn *et al*, 1992; Livingston *et al*, 1994; Hsueh *et al*, 2002; Baars *et al*, 2000). Only few attempts of boosting the immune response to vaccines by the addition of systemic doses of cytokines were made, mostly focused on immunological rather than clinical parameters (Lee *et al*, 2001; Maio *et al*, 2002). The natural candidate for boosting immune responses induced by vaccines is IL-2. Most of the clinical experience with IL-2 as an adjuvant to vaccine was gained with high-dose schedules (Rosenberg *et al*, 1999) or when tumour cells were transduced to secrete IL-2 (Osanto *et al*, 2000). The paucity of clinical combination studies is surprising, in view of murine models that strongly support their rationale (Schrayer *et al*, 2002; Overwijk *et al*, 2003).

The present communication aims at reporting the outcomes of such combination treatment. It is not a controlled clinical trial; it is rather observations that nevertheless can shed some light on the clinical value of combining autologous melanoma vaccine with IL-2.

## PATIENTS AND METHODS

### Patients

The series includes 34 American Joint Committee on Cancer (AJCC) stages III and IV melanoma patients with nonresectable disease that were treated with an autologous melanoma cell vaccine, conjugated with dinitrophenyl (DNP) and mixed with BCG, at Hadassah University Hospital during the years 1996–2003 (Table 1

**Table 1:** Patients' characteristics

*Gender*	
Men	20
Women	14
	
*Age (years)*	
Median	48.5
Range	11−75
	
*Stage*	
III	13
IV	21
	
*Site of metastases*	
Skin and s.c. alone	10
LNs±Skin and s.c.	8
Visceral	16
Lung	9
Liver	6
Bone	2
Ovary	1
Adrenal	1
Patients with more than one site^a^	10
	
*Treatment prior to vaccination*	
ILP	10
Chemotherapy	14
Irradiation	14
IFN*α*	3

s.c.=subcutaneous; LN=lymph node; ILP=isolated limb perfusion; IFN*α*=interferon alpha.

a Including multiple visceral sites or visceral organ with s.c. or LN metastases.

).

All patients were evaluated prior to their inclusion with a total body CT scan. In order to be eligible for the vaccine, patients had to have undergone surgical removal of one tumour deposit and have a Karnovsky performance status of ⩾60%. Exclusion criteria included brain metastases and the systemic use of corticosteroids. Radiation and chemotherapy were not excluding criteria, as long as 6 weeks had elapsed from the last treatment. Indeed, 22 of the patients had undergone prior treatments, including isolated limb perfusion (ILP), chemotherapy, irradiation or combination of several of them. All these patients had documented tumour progression following their last therapeutic regimen. Informed consent was obtained from all patients.

Site of metastates included visceral sites (16 patients), dermal and s.c. tissue alone (10 patients), lymph nodes (3), lymph nodes and s.c. tissue (5). In all, 10 patients had more than one metastatic site.

### Treatment framework (Figure 1)

**Figure 1:**
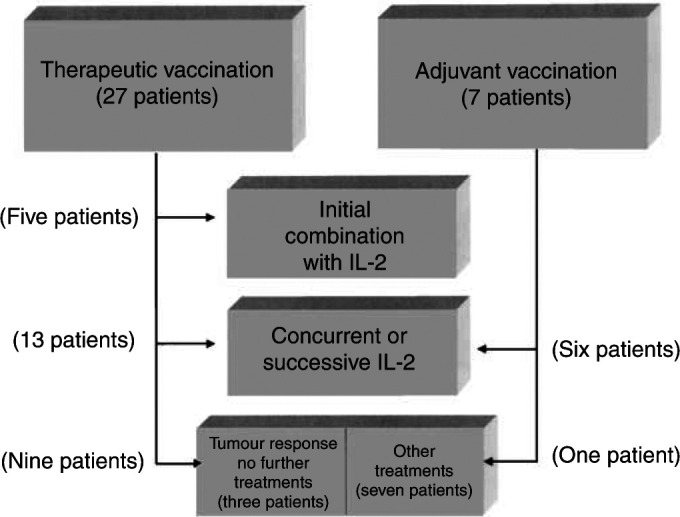
Framework of treatment.

In total, 27 patients were diagnosed with nonresectable, active metastatic melanoma on entry to the vaccination protocol. The other seven patients were free of active disease at that time and received the vaccine as an adjuvant treatment. They developed, however, recurrence in the midst of the adjuvant protocol (between vaccines 4 and 8; three patients) or within 2 years from its initiation (four patients). These seven patients were described elsewhere before (Lotem *et al*, 2002). They are included in the present series because they continued vaccination in the presence of active metastatic disease or were referred for successive IL-2 therapy.

Interleukin-2 was offered to all patients who had no tumour regression after the initial four doses of the vaccine alone, as concurrent treatment and to the patients who had recurrence following the adjuvant autologous vaccine. Five patients with metastatic disease received the combination right from start, as initial vaccine and IL-2. Overall, 24 patients received a combination of vaccine plus IL-2.

### Vaccine preparation

Tumour specimens were procured fresh and sterile. For large tumour bulks, cells were extracted mechanically or by enzymatic dissociation with collagenase and DNAse (Sigma, St Louis, MO, USA), frozen in a controlled rate freezer and stored until needed in liquid nitrogen in a medium containing 2.5% human albumin and 20% DMSO. If the total cell yield was less than 200 × 10^6^ cells, cell suspensions were put into culture bottles with Dulbecco's modified Eagle's medium (DMEM, Gibco BRL, Gaithersburg, MD, USA), 10% fetal calf serum (Gibco BRL), HEPES (1 : 500), pen-strep (1 : 100) and glutamin (1 : 100), and expanded to the required number necessary for preparation of at least eight vaccine doses of 10–25 × 10^6^ cells each. Culturing the cells resulted in the preferential selection of melanoma cells, so that the vaccine finally consisted of purified melanoma cells. Positive staining of more than 50% of cells with at least one of the monoclonal antibodies S-100 and HMB-45, using the alkaline phosphatase antialkaline phosphatase (APAAP) reaction (Zymed Inc, San Fransisco, CA, USA), was required.

On the day of treatment, the cells were thawed, washed and irradiated to 110 Gy. A sample was stained with trypan blue and counted after irradiation. Conjugation of melanoma cells with DNP was performed by the method of Miller and Claman (1976). Briefly, melanoma cells were washed with Hank's balanced solution–no HSA (human serum albumin), resuspended to a concentration of 5 × 10^6^ ml^−1^, mixed, incubated for 30 min and washed again. Prior to vaccination, an appropriate amount of BCG was added, starting with a dilution of 1 : 50 and reaching 1 : 500–1000, according to the resulting granuloma at vaccination site. Each vaccination dose was composed of 10–25 × 10^6^ melanoma cells.

### Vaccination procedure

On Day 1, patients received 300 mg m^−2^ of i.v. cyclophosphamide. On Days 4 and 5, patients were sensitised to DNP by applying 0.1 ml of 2% DNP dissolved in acetone-corn oil (Sigma) topically to the inner aspect of the arm. On Day 18, patients received a second dose of 300 mg m^−2^ cyclophosphamide. On Day 21, the prepared vaccine was injected into three adjacent sites on the upper arm or thigh, avoiding limbs where lymph node dissection had been performed previously. Seven additional doses of the vaccine were administered at intervals of 21–28 days. Before injecting the second dose of the vaccine, a third dose of 300 mg m^−3^ of cyclophosphamide was administered i.v.

Responding patients received additional doses of vaccine with increasing intervals of 3, 3, 6 and 6 months.

### IL-2 schedules

Interleukin-2 was administered in two different protocols: an ambulatory low-dose protocol and an inpatient high-dose protocol.

IL-2 schedules were as follows:

1. Low-dose, s.c. IL-2, given Days 1–5 every 14–21 days, reduced to 3 MIU day^−1^ in patients over 70 years (two patients).
2. High-dose IL-2, consisting of a 2-week course of therapy with a 1-week rest interval in between. Patients were scheduled to get up to 14 touching i.v. doses of IL-2 on each admission, 720 000 IU kg^−1^ of IL-2 per dose, in a 15 min rapid infusion. Evaluation based on physical examination and imaging analysis was performed 6–8 weeks after the first course. Two patients received a second 2-week course. The actual number of doses ranged between 7 and 14, with an average of 10.4 doses per course.

An overall of 24 patients received a combination of vaccine plus IL-2. The first six patients were referred to the high-dose IL-2 protocol. The successive 18 received the low-dose protocol from the start. Two of the low-dose patients who had a transient stable disease and then progressed were referred to the high-dose protocol. One of them died 10 days after treatment, with the clinical presentation of Guillain Barré syndrome. Four other patients with lung metastases received daily inhalations with IL-2 9 × 10^6^ IU dose^−1^ × 2 day^−1^, in addition to their s.c. injections of low-dose IL-2.

Patients who received the combination therapy of vaccine with initial or concurrent low-dose IL-2 were given IL-2 48 h following vaccination. The first two vaccination doses were always administered without IL-2.

Patients were evaluated periodically every 2 months. These evaluations included a physical examination, a complete blood count and liver function tests. A total body CT scan was performed every 2–3 months for the first 2 years, and thereafter at longer intervals.

### DTH evaluation

**Figure 2:**
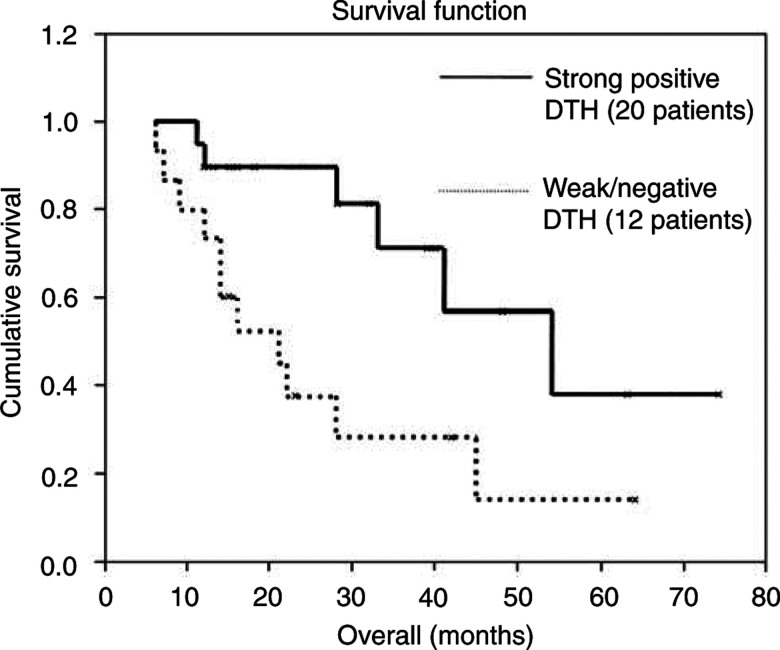
Kaplan–Meier survival curve as a function of DTH response to unmodified autologous tumour cells. Data available from 32 patients. DTH test was not performed for two patients with a rapidly progressing disease.

Skin testing to evaluate delayed-type hypersensitivity to autologous melanoma cells was performed by the intradermal injection of 1–3 × 10^6^ unmodified melanoma cells irradiated to a dose of 170 Gy. To avoid contamination with foreign proteins and chemicals, the cells were triple washed before injection. DTH was measured by the maximal diameter of erythema at 48 h after the injection of the vaccine. Erythema smaller than 5 mm was defined as a negative reaction. We arbitrarily chose the value of 10 mm of erythema to discriminate between weak DTH (<10 mm) and strong DTH (⩾10 mm). The skin test was performed on vaccine doses 4 and 8. Patients were also skin tested to 3 × 10^6^ unmodified autologous lymphocytes (Figure 2

).

### Definition of clinical response

Complete response (CR) was defined as a complete tumour disappearance on imaging analysis or by direct palpation of skin lesions, lasting for at least 3 months.

Partial response (PR) was defined as a reduction of 50% or more of the sum of the greatest perpendicular dimensions of all measurable lesions with no new lesions appearing elsewhere. Stable disease was defined as a regression of less then 50% or increase of no more than 25% in tumour size.

**Figure 3 fig3:**
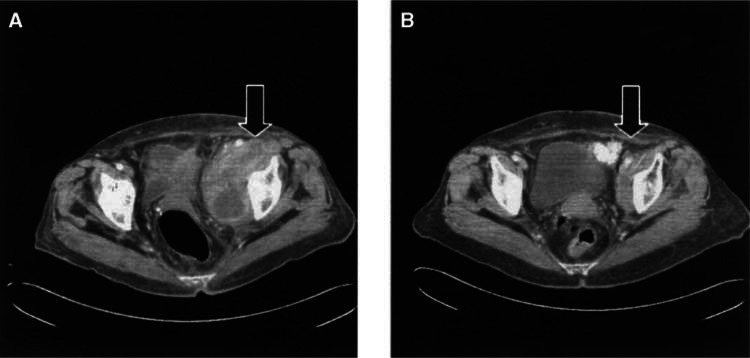
**(A)** Pelvic CT prior to initiation of low-dose IL-2, in a patient who received adjuvant autologous melanoma vaccine, and had recurrent pelvic LN metastases 12 months later (patient 3). **(B)** Partial regression of pelvic lymph nodes after 6 months of therapy.

In two cases, we were unable to review the CT scans and had to rely on a formal radiological report, comparing two successive analyses, carried out 3 months apart, at another medical center (cases 7 and 14, Table 3)

.

### Statistical analysis

Survival time was calculated from the date of surgery to the date of either last analysis or death. Duration of response was defined from first vaccine or from first course of IL-2, depending on the therapy that yielded the response.

The overall survival (OS) was estimated by the Kaplan–Meier's method. The impact of DTH response to autologous tumour cells on OS was evaluated. Significance was calculated by the log-rank test.

## RESULTS

### Survival analysis

In all, 34 patients are included in this series, with a median follow-up of 21.5 months (range 4–74 months). Using the Kaplan–Meier method and the log-rank test, the OS rate of the whole group is 40 months (standard error 5.0; 95% confidence interval 30.5–50.3 months). Patients who attained a strong positive DTH to their autologous tumour cells had an OS from time of diagnosis of 54 months (standard error 13; 95% confidence interval 28–79 months) compared to 21 months (standard error 5; 95% confidence interval 16–37 months) for those who had weak or negative DTH, a difference found significant (*P*=0.013, see Figure 2). However, the difference may have been even larger, since there are two patients with weak DTH who have exceptional long-term survival that skewed the data of the whole group.

### Response rates (Table 2)

**Table 2:** Response rates

	**CR**		**PR**		**Stable**		**Progressing**	
	***N***	**%**	***N***	**%**	***N***	**%**	***N***	**%**
Total (34 patients)	4	11.8	8	23.5	5	14.7	17	50.0
Vaccine only^a^ (22 patients)	2	9.1	0	—	1	4.5	19	86.4
Vaccine+IL-2	2	9.1	8	36.4	2	9.1	10	45.5
Low dose (17 patients)	1	5.9	6	35.3	2	11.8	8	47.0
High dose (seven patients)	1	20.0	2	40.0	0	—	2	40.0

a The effect of vaccine treatment alone, before adding IL-2. CR=complete response; PR=partial response; IL-2=Interleukin-2.

Only two out of the 22 patients who went through a phase of vaccine alone (other seven were adjuvant patients and five received a combination from start) had demonstrated objective regressions of their tumours (9%). Both had complete disappearance of s.c. metastases after they had progressed following ILP. In addition, one of these two had previously received chemotherapy with DTIC and cisplatinum as well as local irradiation to the skin, without success.

In contrast, 10 of 24 patients showed complete or partial regression of their tumours, following the phase of IL-2 administration. The median duration of regression period was 6 months (mean duration of 14 months), ranging from 3 to 50 months. Two patients achieved CR, one of them is still in CR for 50 months, the other recurred after 14 months. Of the eight patients with PR, two still go on (4+ and 15+ months). Four of the responders had their IL-2 combined with the vaccine from the beginning. Three of the responders were participants of the adjuvant study that recurred within 12–24 months and were then included in the low-dose IL-2 regimen. The other three experienced disease progression during the course of vaccination, between vaccine doses 4 and 8, and were referred to high-dose IL-2. The patient with the long-term CR is among them.

### IL-2 schedules

Of the 17 patients who received low-dose IL-2, six had PR and one had CR, a response rate of 41%. In comparison, three of the patients who received high-dose IL-2 showed responses (43%): one CR, two PRs lasting for 3 and 11 months. However, the number of patients is too small to detect significant differences of RRs. Notably, the tumour shrinkage induced by IL-2 was a lengthy process that continued months after the last course of high-dose IL-2, with no additional treatment. In patients receiving low-dose IL-2, therapy was continued for as long as the response was maintained. Patient 4 is treated with IL-2 for the longest period, 15 months now and still demonstrates slow tumour regression. Table 3

**Table 3:** Clinical data of patients achieving objective responses

**Pt #**	**Sex**	**Age (year)**	**Previous treatments for metastatic disease**	**Vaccine setting**	**IL-2 protocol**	**Interval between vaccine and IL-2**	**Site of metastases**	**Type of response**	**Site of response**	**Duration (months)**
1	F	69	Surgery, ILP, DTIC+cDDP, XRT	Metastatic	No IL-2		Skin, leg and thigh	CR	Skin, leg and thigh	42+
2	F	75	Surgery, ILP, XRT	Metastatic	No IL-2		Skin, leg	CR	Skin, leg	32+
3	F	71	Surgery, DTIC, XRT	Adjuvant	Low dose	12 mo	Pelvic adenopathy	PR	Pelvic adenopathy(Figure 3)	10
4	M	58	Surgery, ILP, XRT	Metastatic	Low dose	Concurrent	Skin, leg; inguinal LN	PR	Skin, inguinal LN(Figure 4)	15+
5	F	55	Surgery, DTIC+cDDP+low-dose IL-2+GM-CSF, XRT	Metastatic	Low dose	Concurrent	Brain resected; paravertebral mass; lung	PR	Lung, paravertebralmass	3
6	F	44	Surgery, adjuvant IHA fotemustine, radiofrequency ablation	Adjuvant	Low dose	24 mo	Liver	PR	Liver	6
7	M	57	Surgery	Metastatic	Low dose+inhalation	Concurent	Lung, mediastinal LN	PR^a^	Lung	3
8	M	68	Surgery	Adjuvant	Low dose+inhalation	12 mo	Lung	PR	Lung(Figure 5)	4+
9	F	55	Surgery, single dose of BCG	Metastatic	Low dose+inhalation	Concurrent	Skin, thigh resected; seven bilateral lung mets	CR	Lung	14
10	M	52	Surgery, chemotherapy (cDDP+DTIC, fotemustine), pelvic perfusion with cDDP	Metastatic	High dose	20 mo	Skin, lower extremity; pelvic LNs	PR	Skin, lower extremity; pelvic LNs	11
11	F	28	Surgery, LD IFN, ILP with melphalan	Metastatic	High dose	After eight vaccinations	Skin, lower extremity; pelvic LNs	CR	Skin	50+
12	F	54	Surgery	Metastatic	Low dose and later high dose	Concurrent	Skin, lower extremity; lung	PR^a^	Lung	3

cDDP=cisplatinum; XRT=radiotherapy; IHA=intrahepatic artery infusion; HD=high dose; LD=low dose; IL-2=Interleukin-2; ILP=isolated limb perfusion; GM-CSF=granulocyte–macrophage colony-stimulating factor; IFN=interferon; mo=months; LN=lymph node, CR=complete response; PR=partial response.

a Based on CT report of another medical center; DTIC=dacarbazine.

provides detailed clinical data on responding patients.

Seven patients who progressed during their vaccination protocol were not referred to receive IL-2: four because of rapidly progressing disease, one who refused and two patients who achieved CR following local irradiation of their tumour masses.

### DTH and tumour regression

Strong inflammatory reactions to autologous tumour cells evaluated by the DTH test were evident in 20 patients (59%). Of them, 11 (55%) attained a response to either vaccine alone (2) or the combination (9). One responder had a DTH test of only 6 mm. In comparison, none of the 12 patients who failed to show a positive DTH had any objective tumour regression. DTH data are missing for two patients, who had a rapidly progressive disease.

### Site of regressing metastases

In all, 12 patients had objective tumour regressions. These regressions involved dermal and s.c. metastases in seven cases, lung metastases in four, lymph node metastases in four and bone in one.

### Autoimmune phenomena

**Figure 4:**
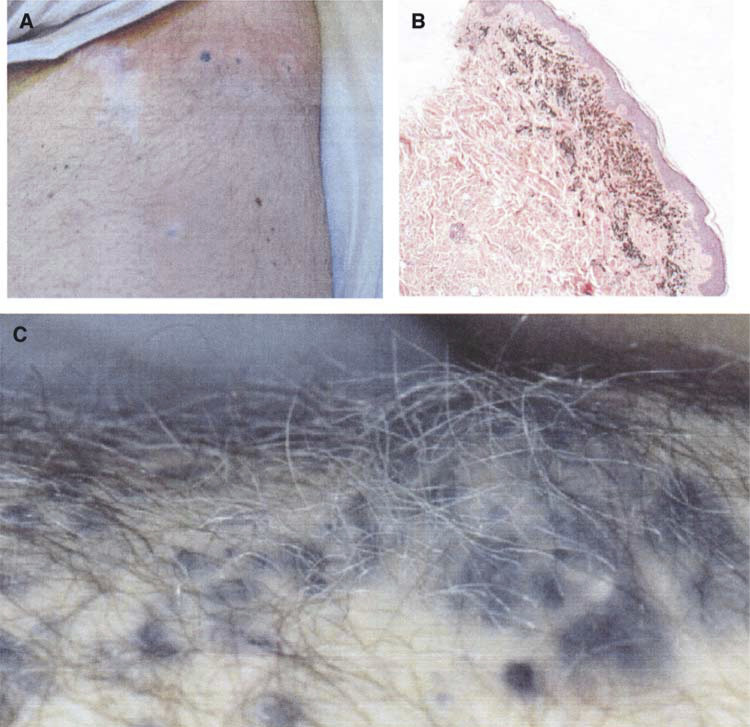
**(A)** Regressing dermal metastases with perilesional vitiligo in patient 4. **(B)** Melanin-laden melanophages in upper dermis, in a skin biopsy from a regressing lesion of patient 4 (H&E × 20). **(C)** White hair arising from regressed s.c. metastases. Hair of noninvolved skin is fully melanised (patient 11).

Two patients, both in whom tumour regressions occurred, developed vitiligo. The vitiligo was most pronounced at the area of regressing metastatic deposits (Figure 4

). In one patient, persistent elevated CPK levels were measured without electromyographic abnormalities. This patient had undergone ILP prior to his immunisation.

## DISCUSSION

The data presented here show an OS of 40 months after autologous vaccine therapy of patients with metastatic melanoma. The objective tumour regression to the vaccine, however, was minimal. Only in two out of the 34 patients this regression was measurable. These findings correspond with previous publications by the John Wayne Cancer Institute (Hsueh *et al*, 2002), using an allogeneic melanoma vaccine.

Yet, our retrospective series shows that upon the addition of IL-2, RR rose to 42%. This considerable additional therapeutic benefit suggests that the vaccine enhances the effect of IL-2 and increases the RR to this cytokine. Any statements based on a small retrospective series are limited, but the reported responses to IL-2 therapy alone are in the order of 15–20% (Atkins *et al*, 2000), and are particularly lower when low-dose IL-2 is administered (Barth and Mule, 2000). It appears, therefore, that the autologous vaccine exerted a priming effect, possibly generating tumour-specific immune effector cells, which were activated upon the addition of IL-2.

The administration of an autologous melanoma vaccine was shown to be associated with the expansion of particular T-cell clones within the tumour as well as in peripheral blood. This was measured by post-therapy increment in antitumour cytolytic activity, by increased cytokine production of lymphocytes to tumour antigens and by rearranged TCR repertoire, which indicates the expansion of specific clones, in peripheral blood or within the tumour (Soiffer *et al*, 1998; Belli *et al*, 2002; Krause *et al*, 2002; Manne *et al*, 2002). DTH reaction to tumour cells is a useful clinical test that reflects cell-mediated antitumour immunity. Both CD4+ and CD8+ melanoma-reactive T-cell subsets were isolated from skin biopsies of DTH sites (Waanders *et al*, 1997). Out of the 12 responding patients in the present series, 11 had developed a strong positive DTH response to their autologous tumour cells. None of the patients who failed to demonstrate a positive DTH response had any objective tumour regressions, even with the addition of IL-2. In a previous study, we found a significant correlation between the development of strong positive DTH and survival (Lotem *et al*, 2002). Similar findings were reported by Berd *et al* (1990, 2001) and Baars *et al* (2000). The results presented here suggest that this is not only a prognostic marker but may also be a prerequisite for tumour regression, attesting to the presence of tumour-specific T cells. These preformed effectors are probably the key to the enhanced effect of IL-2.

Interleukin-2 is a potent T-cell growth factor. This function of IL-2 is the basis for the adoptive transfer of *ex vivo*-activated T cells. High doses of IL-2 are required to sustain the growth and proliferation of PBMC-derived tumour-reactive T cells and of clonal tumour infiltrating lymphocytes that were expanded *ex vivo*, after their reinfusion (Rosenberg *et al*, 1988; Dudley *et al*, 2002). Immunisation with an immunodominant peptide derived from gp100 antigen prior to the administration is a different, *in vivo* approach to generate and expand peptide-specific T cells. This regimen was associated with a 42% RR (Rosenberg *et al*, 1998). The relatively high RR we have witnessed when IL-2 was given following immunisation suggests that the whole tumour vaccine may have generated tumour-reactive T-cell subsets. Interleukin-2 was probably required to turn these precursors into effector cells. In spite of the fact that the series presented here is far too limited to indicate advantage of low *vs* high doses of IL-2, it is nevertheless encouraging to note that the less toxic protocol brought about several prolonged responses.

A majority of the patients who responded by tumour regressions have had s.c. or pulmonary deposits. The increased susceptibility of skin and lung metastases to immunotherapy has been addressed before (Berd *et al*, 2001; Hsueh *et al*, 1999). Interestingly, several of our responding patients had received cytodestructive modality previously: ILP or radiotherapy, to which they responded by transient initial response. We assume that a treatment associated with cell damage might have exposed the patients to release tumour antigens. This constitutes the basis for combining chemotherapeutic agents with IL-2, which clearly has no direct antitumour cytotoxicity. The combination results in higher RRs (Eton *et al*, 2002).

**Figure 5 fig5:**
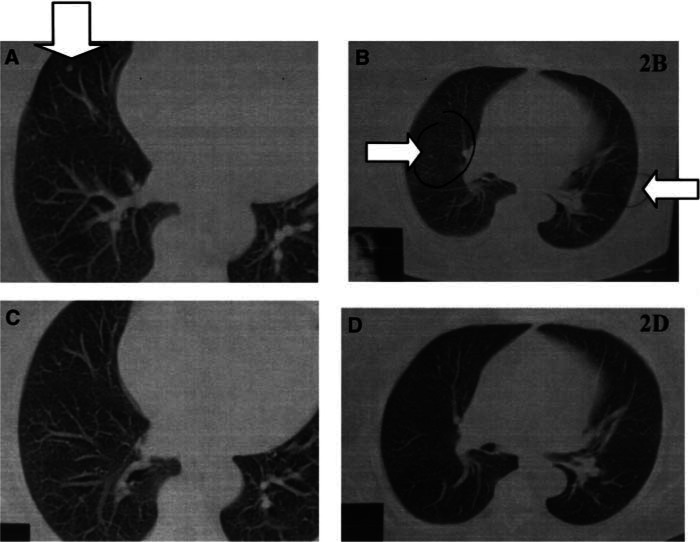
Patient 9: CT of the thorax. **(A** and **B).** Three metastatic deposits <1 cm. (**C** and **D**). At 3 months after initiation of autologous melanoma vaccine with low dose and inhalations of IL-2–significant partial regression of all lesions. Patient has eventually achieved CR.

It is impossible to draw decisive conclusions based on a small retrospective series. Yet, the RR to combined vaccine and IL-2 treatment reported here is much higher than the known effect to the cytokine therapy alone, which cannot be overlooked. Patient selection for these protocols can be based on the location of their metastatic deposits, preferring lung and skin, as well as on positive DTH to autologous melanoma cells. Clinicians who practice antitumour vaccination may wish to try to proceed with an IL-2-based regimen in the appropriate patients[Fig fig5]

.
